# Effects of Discharge Parameters on the Thawing Characteristics and Physicochemical Properties of Beef in a Dielectric Barrier Discharge (DBD) System

**DOI:** 10.3390/foods13213360

**Published:** 2024-10-23

**Authors:** Jie Zhang, Rui Zhao, Yaming Zhang, Huixin Wang, Zhiqing Song, Ru Xing, Jingli Lu, Changjiang Ding

**Affiliations:** 1 College of Electric Power, Inner Mongolia University of Technology, Hohhot 010051, China; 202211000700@imut.edu.cn (J.Z.); zqsong@imut.edu.cn (Z.S.); 2 College of Science, Inner Mongolia University of Technology, Hohhot 010051, China; 20201100076@imut.edu.cn (R.Z.); 20181100063@imut.edu.cn (Y.Z.); 20211100068@imut.edu.cn (H.W.); 3 School of Physical Science and Technology, Baotou Teacher’s College, Baotou 014030, China; meiyingyy@126.com

**Keywords:** dielectric barrier discharge (DBD), discharge parameters, thawing beef, thawing characteristics, water-holding capacity (WHC), Fourier transform infrared (FTIR)

## Abstract

Traditional thawing techniques can cause certain losses to beef quality. Due to the increasing demand for high-quality beef, there is an urgent need to research new thawing techniques. Dielectric barrier discharge (DBD), as an innovative non-thermal thawing technology, still has a lot of work to be studied. In order to explore the influence of DBD on the thawing characteristics and quality of beef, different discharge parameters were used for thawing. The results show that voltage and needle distance have significant effects on ion wind speed and composition. Ion wind can improve the thawing rate, and the thawing time of DBD is 50% shorter than that of natural thawing. DBD improved the water-holding capacity, nutritional components, and color of beef, and the ordered structure of beef protein could be improved by 6.25% at most. The plasma emission spectrum shows that the plasma produced by DBD is mainly active substances of nitrogen and oxygen, which can reduce the fat oxidation of thawed beef and improve the quality of beef. This work provides the theoretical basis and practical guidance for deeply understanding the influencing parameters and thawing mechanism of DBD thawing technology.

## 1. Introduction

Inner Mongolia Autonomous Region’s vast grassland breeds abundant beef resources in China. Beef is not only delicious but also contains many valuable ingredients beneficial to human health, such as taurine, carnosine, 4-hydroxyproline, goose serotonin, and creatine. These components show excellent biological activity in the human body, which can effectively resist oxidative stress and inhibit inflammatory reactions, thus playing a positive role in improving tissue damage and cardiovascular dysfunction [1]. For this reason, beef has increasingly become the focus of public attention, and its nutritional value and health effects are highly respected.

Frozen storage is recognized as an efficient and high-quality storage method in the meat industry, and its thawing link is a prerequisite for meat products to enter the consumption and processing chain. However, improper thawing methods often lead to a series of problems, including the loss of nutrients, the intensification of lipid and protein oxidation, the deterioration of color and flavor, and the growth of microorganisms, all of which pose a significant threat to economic benefits [2]. Traditional thawing techniques, such as air thawing and hydrolysis thawing, are widely used. Still, they are generally faced with the challenges of low thawing efficiency and great quality loss and are easily troubled by microbial pollution. In recent years, with the progress of science and technology, emerging technologies such as microwave thawing [3], vacuum thawing, and ultrasonic thawing [4] have been proven to significantly shorten the thawing cycle and effectively improve the final quality of products. However, each technology has its own unique limitations, and it is difficult to fully meet the diversified needs of industrial production. Vacuum thawing technology requires a large number of field personnel and expensive equipment. In ultrasonic thawing technology, increasing ultrasonic intensity can effectively shorten the thawing time, but the cavitation effect may destroy the cell structure and increase the nutritional loss of the product [5]. Using thermal effect, microwave thawing technology is prone to uneven heating and local overheating, which leads to partial thawing of products and reduces product quality [6]. Therefore, continuous exploration and innovation to find a more efficient, safe, and environmentally friendly thawing method is of great significance in promoting the healthy development of the meat processing industry.

High-voltage electric field (HVEF) thawing technology, as an innovative thawing method, is being widely concerned and deeply studied. The research shows that the HVEF shows double advantages during the thawing process: it can not only significantly improve the thawing efficiency but also have a significant sterilization effect, effectively reducing the concentration of microorganisms [7]. In addition, the further exploration of Rahbari et al. [8] reveals another important feature of HVEF—it can not only effectively reduce the number of microorganisms but also significantly delay the degradation process of ATP, which is very important for maintaining the freshness and safety of food. Wang et al. [9] applied the high-voltage electric field thawing technology to the thawing process of hawthorn. They found that this method not only greatly shortened the thawing time but also significantly improved the quality of thawed hawthorn. HVEF defrosting technology adopts a plate-to-plate structural design. In this process, the air is ionized under the action of the electric field, and the generated charged particles move to the negative electrode driven by electric field force, thus forming a breakdown phenomenon. In contrast, dielectric barrier discharge (DBD) technology adds a dielectric barrier to the board-to-board structure, which effectively blocks the movement of charged particles, thus avoiding breakdown. At the same time, DBD technology can produce more abundant ions and stronger ion wind speed, which provides more diversified possibilities for food processing. To sum up, HVEF thawing technology has shown great potential in the field of food processing with its unique advantages. The dielectric barrier discharge (DBD) processing technology developed on this basis further broadens the application prospect of food processing technology.

Dielectric barrier discharge (DBD) is an innovative non-thermal technology that uses a specially designed electrode system to cover one of the electrodes with a dielectric and apply a high voltage difference to work [10]. DBD is a new non-thermal technology widely used in food processing. Roobab et al. [11] found that DBD can be used as an effective non-thermal purification method in meat processing, and the ozone, plasma, and high-voltage electric fields generated in the process have a powerful killing effect on microorganisms. Wang et al. [12] studied the effect of dielectric barrier discharge atmospheric cold plasma on *Bacillus acidophilus* and found that DBD could effectively inactivate the acid soil mold by significantly increasing the permeability of the cell membrane, the leakage of cytoplasmic contents, and the change in bacterial morphology. Umair et al. [13] believed that the number and abundance of active species during dielectric barrier discharge played an important role in spore inactivation. Makari et al. [14] treated *Aspergillus flavus* spores with DBD; the number of viable spores decreased significantly with the extension of DBD treatment time, and no detectable spores were found after 180 days of treatment. Roy et al. [15] used DBD for the periodic processing of food, which can extend the shelf life of products. Hu et al. [16] used a DBD device to inactivate *E. coli*, and they found that extracellular antibiotic resistance genes (e-qnrB, e-blaCTX-M, e-sul2) and integrator genes (e-int1) decreased by 1.99, 2.22, 2.66, and 2.80 logs, respectively, in the first 5 min of discharge. Tang et al. [17] found that when the input power of DBD exceeds 100 W, the number of live viruses in the exposure time of 30 s decreased by 1.3 log values, and the number of live viruses in the exposure time of 120 s decreased by 5.3 log values. To sum up, the research on DBD in the food field mainly focuses on decontamination, sterilization, food packaging, and drying [18], but the research on food thawing is relatively few, and further exploration is needed in the future.

In this research, beef was thawed by DBD with different voltages and needle spacings. The water-holding capacity and color of the beef were measured. The beef was also scanned by infrared spectroscopy, and the secondary structure of the protein was calculated to explore the effect of DBD thawing on beef quality. This study provides a theoretical and experimental basis for the application of DBD to thawing.

## 2. Materials and Methods

### 2.1. Experimental Materials and Device

The fresh beef was purchased from the same supermarket. After preliminary research, it was found that the weight range of thawed meat samples in the laboratory stage is mostly between 20 g and 100 g [19,20,21,22,23]. So, cut the beef into 99 square samples of 30 mm × 30 mm × 35 mm; each piece of beef weighs 41 ± 1 g, and store all the samples in the refrigerator at −20 °C for 24 h to prepare for the thawing experiment. Three beef samples were randomly selected from each group for the thawing experiment, which was repeated at least three times.

The experimental device is mainly composed of a high-voltage power supply, voltage controller (YD(Z)-1.5/50, Wuhan, China), needle electrode plate, dielectric barrier plate, and grounding plate electrode. The controller outputs AC of a high voltage power supply with a frequency of 50 Hz. The needle electrode plate is composed of an iron needle with a length of 30 mm and a width of 1 mm. The grounding plate is an 85 cm × 40 cm iron plate; the distance between the needle tip and the grounding plate is 10 cm; and the dielectric barrier plate is a 100 cm × 60 cm, 5 mm thick polyvinyl chloride (PVC) board. This device generates plasma by ionizing the air through the discharge of the tip of the needle electrode plate.

### 2.2. Experimental Method

**Figure 1:**
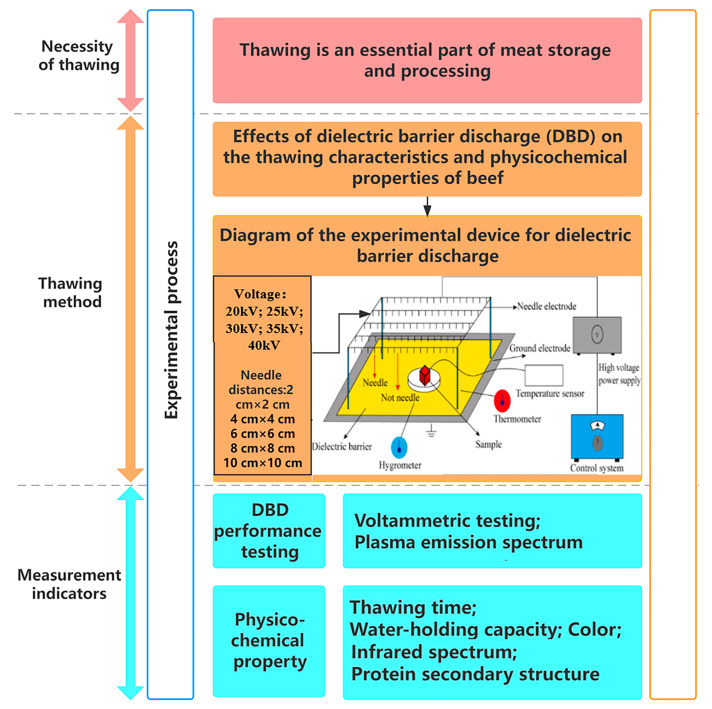
Schematic diagram of the experimental process and key steps.

A natural thawing experiment (the control group) was carried out at a temperature of 25 ± 2 °C, relative humidity of 38 ± 2%, and ambient wind speed of 0 m/s. Through the preliminary experimental results, it can be found that the thawing effect under different voltages and different needle distances is different, and the quality of thawed beef is also obviously different. Therefore, the experimental conditions of different voltages and different needle distances are used to explore the thawing law of DBD. The thawing conditions of DBD are as follows: (1) The needle distance (the distance between two adjacent needles) is 4 cm × 4 cm, the polar distance is 10 cm, and the voltages are 0 (natural thaw), 20, 25, 30, 35, and 40 kV; (2) The voltage is 40 kV, the polar distance is 10 cm, and the needle distance is 2 cm × 2 cm, 4 cm × 4 cm, 6 cm × 6 cm, 8 cm × 8 cm, and 10 cm × 10 cm. The temperature measuring end of the digital temperature sensor is inserted into the beef, and the central temperature of the beef is continuously monitored and recorded. In order to ensure the accuracy and reliability of the experiment, the central temperature of beef reached −10 °C as the starting point of the thawing experiment, and the central temperature was recorded every 5 min. When the central temperature reaches 0 °C, the thawing experiment ends. The schematic diagram of the experimental process and key steps is shown in Figure 1.

### 2.3. Measurement of Electric Field Discharge Characteristics

Ionic wind speed was measured using a thermal anemometer probe. Discharge images were acquired using a camera (Nikon D7000, Tokyo, Japan) with an exposure time of 40 ms. DBD emission spectra were obtained using a spectrometer and an ICCD camera (DH334T-18U-E3, Beijing, China).

### 2.4. Specific Energy Consumption

The specific energy expenditure of beef during the thawing process was calculated using the method of He et al. [7]. The current is obtained by measuring the current between the grounding plate and the floor using a multimeter. The formula is as follows:

(1) SEC=(VIt)/m

where *V*, *I*, and *t* and *m* are voltage (kV), current (mA), thawing time (s), and mass of beef (kg), respectively.

### 2.5. Water-Holding Capacity (WHC)

Water-holding capacity (WHC) is an important parameter for evaluating meat quality. It refers to the ability of the muscle to keep its original moisture when it is subjected to external forces (such as pressure, heating, cutting, thawing, and other processing or storage conditions). It is usually evaluated by thawing loss, centrifugation loss, and cooking loss [16].

#### 2.5.1. Thawing Loss Determination

The thawing loss was calculated using the approach of Xia et al. [24]. The mass of the beef samples was measured before the thawing experiment. After thawing, the surface juice was dried with qualitative filter paper, and the mass of beef samples was measured. The thawing loss rate was calculated as follows:

(2) $$Thawing\;loss=(m_{1}-m_{2})/m_{1}\times 100\%$$

where *m*_1_ and *m*_2_ are the mass of the beef before and after thawing, respectively.

#### 2.5.2. Centrifuge Loss Determination

The measurement method of Bian et al. [25] was slightly modified. The thawed beef strips, each with a mass of 2 ± 0.1 g, were put into a centrifuge tube and centrifuged at 4000 r/min for 40 min using a low-speed bench centrifuge. After centrifugation, the surface moisture was dried with qualitative filter paper, and the quality of the centrifuged beef was measured. The centrifugation loss rate was calculated as follows:

(3) $$Centrifuge\;loss=(m_{a}-m_{b})/m_{a}\times 100\%$$

where *m_a_* and *m_b_* are the mass of the beef before and after centrifugation, respectively.

#### 2.5.3. Cooking Loss Measurement

The cooking loss was measured with a slight modification to the method of Qian et al. [26]. After thawing, the samples were cut into 30 ± 2 g squares and placed into a 97 °C digital display constant temperature water bath for 40 min. The cooking loss rate was calculated as follows:

(4) $$Cooking\;loss=(m_{3}-m_{4})/m_{3}\times 100\%$$

where *m*_3_ and *m*_4_ are the mass of the beef before and after cooking, respectively.

### 2.6. Color Measurement

The lightness (L*), redness (a*), and yellowness (b*) of the surface of the fresh and thawed beef samples were measured by a chroma meter (3nh-NR60CP, Shenzhen, China). The changes in L*, a*, and b* of beef before and after thawing were calculated. Three measurements were taken for each piece of beef and averaged for the final result. The calculation formula is below [27]:

(5) $$\Delta L^{*}=L_{1}^{*}-L_{0\;}^{*}$$

(6) $$\Delta a^{*}=a_{1}^{*}-a_{0}^{*}$$

(7) $$\Delta b^{*}=b_{1}^{*}-b_{0}^{*}$$

(8) $$\Delta E=\sqrt{{\left(\Delta L^{*}\right)}^{2}+{\left(\Delta a^{*}\right)}^{2}+{\left(\Delta b^{*}\right)}^{2}}$$

where $L_{0}^{*}$, $a_{0}^{*}$, and $b_{0}^{*}$ are the fresh beef brightness, redness and yellowness, respectively. $L_{1}^{*}$, $a_{1}^{*}$, and $b_{1}^{*}$ are the thawed beef brightness, redness and yellowness, respectively.

### 2.7. Fourier Infrared Spectroscopy (FTIR) and Protein Secondary Structure Determination

The method of measurement of the infrared spectrum is described by Cheng et al. [28] with some modifications. The thawed beef samples were finely screened and crushed, and then the obtained powder was fully mixed with potassium bromide. The mixture was placed in a tablet press, and a uniform tablet sample was formed by pressing. In order to improve the purity of spectral data, a Fourier transform infrared spectrometer (IRTracer-100, Shimadzu, Japan) is used to scan the sample, which effectively eliminates interference factors such as water and carbon dioxide and ensures the clarity and reliability of the scanned spectrum.

By selecting the 1600–1700 cm^−1^ band of the infrared spectrum for inverse integration, second derivative, and curve fitting, the percentage of secondary structures in protein is calculated.

### 2.8. Statistical Analysis

In this study, we use Origin (2021) software as a data visualization tool to graphically display the experimental data. To ensure the stability and reliability of the experimental results, three independent repeated operations were carried out for each group of experiments. The final results are presented in the form of mean standard deviation (SD). With the help of the statistical analysis software SPSS (26.0), the significant differences in experimental results are determined by one-way analysis of variance (ANOVA). When *p* < 0.05, it is considered that the observed difference is statistically significant.

## 3. Results and Discussion

### 3.1. Plasma Emission Spectrum

**Figure 2:**
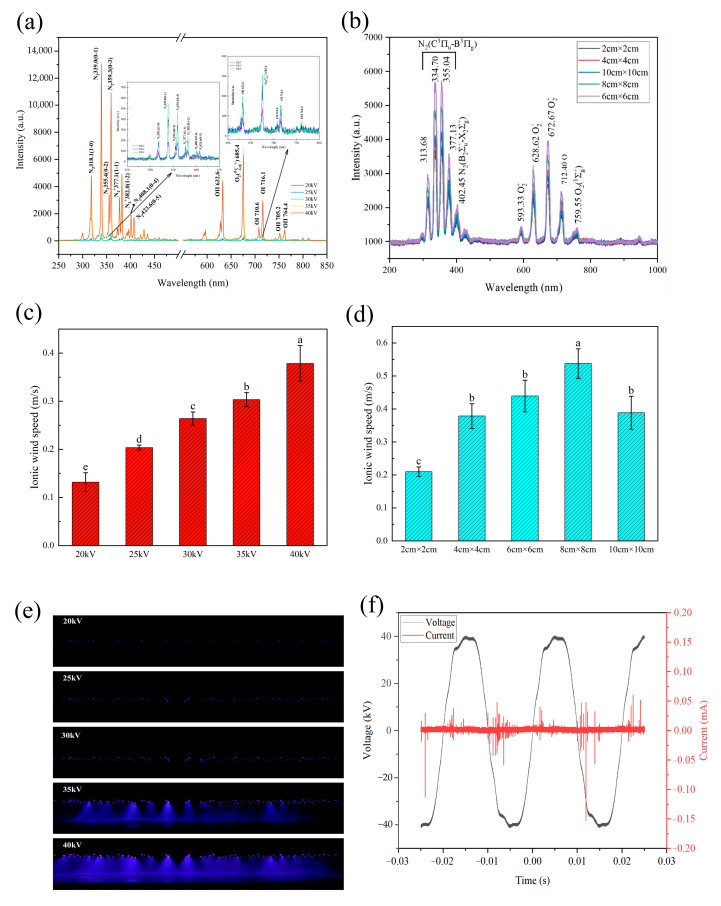
DBD characteristics. (**a**) Emission spectra at different voltages; (**b**) Emission spectra at different needle distances; (**c**) Ion wind speed at different voltages; (**d**) Ion wind speed at different needle distances; (**e**) Discharge pattern at different voltages; (**f**) Voltammetric characteristic diagram. Note: Different letters indicate significant differences (*p* < 0.05) between sample means.

Figure 2a,b show plasma emission spectra at different voltages and needle distances. The substances produced by DBD are mainly active substances such as N_2_, N_2_^+^, O_2_^+^, O, and O_2_, and their contents were significantly different among experimental groups. With the increase in voltage and needle distance, the substance content increased gradually, reached the maximum when the needle distance was 6 cm, and then decreased. In the range of 200–1000 nm, the content of N_2_ (C^3^π_u_-B_3_π_g_) and O_2_^+^ increases significantly, and a variety of reactions will be carried out between the active substances to produce nitroxic compounds, which may affect the quality of beef. At present, there is a wide range of research on food applications. Jung et al. [29] first proposed the use of treated plasma-treated water as a source of nitrite in food and verified it by using plasma-treated water to treat sausage. Later studies found that similar results could be achieved by treating food directly with plasma. Chen et al. [30] directly used atmospheric non-thermal plasma to treat the roast leg of lamb for 45 min, and the nitrite content could reach 1.88 mg/kg and reduce lipid oxidation. However, Jayasena et al. [31] obtained the opposite result by using DBD plasma to treat chicken and beef tenderloin. The active substance increased lipid oxidation, and the degree of oxidation of beef was higher than that of chicken. Figure 2e shows that with increasing voltage, the phenomenon of discharge increases gradually. Figure 2f shows that when the voltage increases, the peak value of the spectrum gradually increases, and some new peaks appear. The change in plasma distribution proves that the velocity of ion wind and the species of ions increase with the increase in voltage and needle distance. When the electric field intensity exceeds the ionization intensity of nearby air, the air floating near the electrode produces partial discharge, i.e., DBD. The DBD can ionize the air to produce increased plasma. Under the action of the electric field, many neutral particles were mixed and blown away from the electrode to form the ionic wind. The research results of Grosse et al. [32] also support the view that ion wind speed increases with voltage. In addition, the in-depth exploration of Hoeft et al. [33] shows that with the increase in voltage, the product mode of DBD gradually changes from O_3_ to N and O modes, which is highly consistent with our current experimental results. It is worth noting that the ablation mechanism of DBD is complicated, and its efficiency is jointly restricted by plasma composition, gas type, power parameters, exposure time, distance between electrode and sample, plasma generation rate and concentration, and inherent characteristics of ablation materials. By comprehensively considering ion wind speed, discharge characteristics, and plasma spectral data, we can more thoroughly analyze the key factors affecting DBD thawing efficiency.

### 3.2. Specific Energy Consumption Analysis

**Figure 3:**
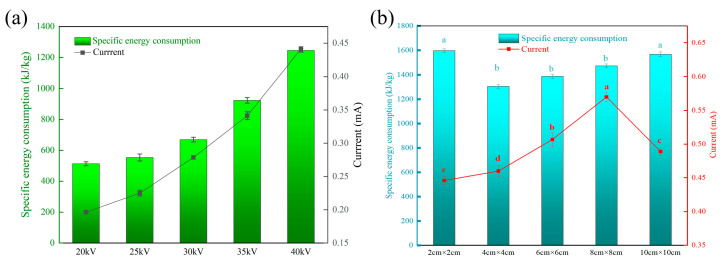
Specific energy consumption and current. (**a**) Specific energy consumption and current at different voltages; (**b**) Specific energy consumption and current at different needle distances. Note: Different letters indicate significant differences (*p* < 0.05) between sample means.

Figure 3 shows the current and specific energy consumption of the DBD system under different parameters. The current in each experimental group is obviously different. It can be seen from Figure 3b that the current first increases and then decreases with the increase in needle pitch and reaches the maximum when the needle pitch is 8 cm × 8 cm. The specific energy consumption of needle pitches of 4 cm × 4 cm, 6 cm × 6 cm, and 8 cm × 8 cm is low, which are 1306.76 kJ/kg, 1388.34 kJ/kg, and 1473.44 kJ/kg, respectively. There is no significant difference between them because the specific energy consumption is affected by voltage, current, and time at the same time. When the voltage is fixed, thawing time and current are the key factors. However, with the increase in needle distance, the current increases and the thawing time shortens, resulting in no difference between the experimental groups. He et al. [7] measured a specific energy consumption of 1104 kJ/kg when using microwave technology to thaw pork, indicating that the energy consumption of DBD thawing technology is not much different from that of microwave thawing technology. When the needle distance is fixed at 4 cm × 4 cm, the current and energy consumption increase with the increase in voltage. Ni et al. [34] found that in the process of plasma drying (taking lotus pollen as an example), the total energy consumption significantly exceeded the net energy consumption, and the difference was as high as three to five orders of magnitude. This discovery highlights the shortage of energy conversion efficiency of high-pressure conversion equipment at present and then points out that inefficient high-pressure conversion technology is still the key factor restricting the wide application and development of DBD dryers in the industry. Therefore, improving the efficiency of high-voltage conversion equipment and reducing unnecessary energy loss will be the keys to promoting the development of DBD thawing technology.

### 3.3. Thawing Time and Central Temperature

**Figure 4:**
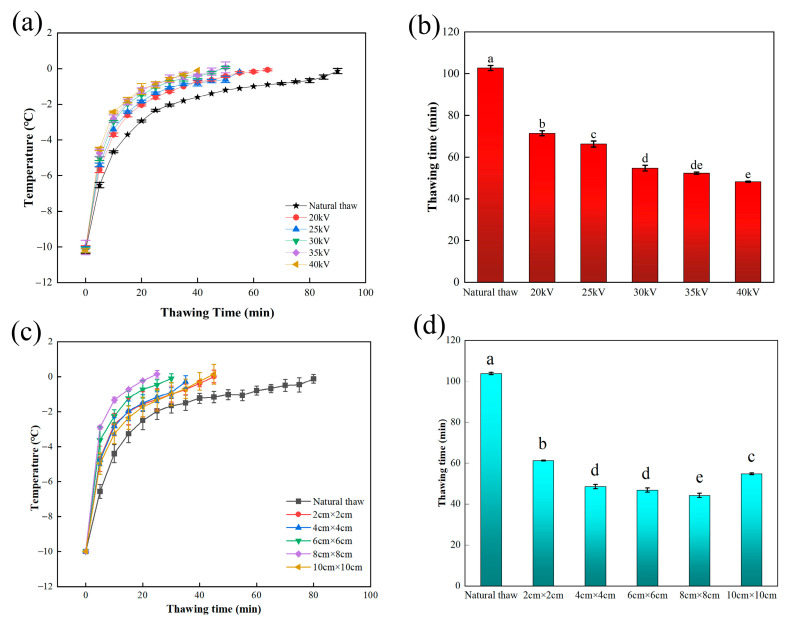
Thawing center temperature and thawing time of beef. (**a**) Center temperature of beef thawing at different voltages; (**b**) Thawing time of beef at different voltages; (**c**) Thawing center temperature of beef under different needle distances; (**d**) Thawing time of beef under different needle distances. Note: Different letters indicate significant differences (*p* < 0.05) between sample means.

Figure 4 shows the central temperature curve and thawing time of the beef thawing process. Under all experimental conditions, the thawing process of beef treated by DBD is obviously faster than that of the natural thawing method. Specifically, when the geometric center temperature of beef is in the range of −10 °C to −5 °C, its temperature rises rapidly. When the temperature further rises from −5 °C to 0 °C, the rising speed tends to be flat. It is particularly noteworthy that at this critical stage from −5 °C to 0 °C, DBD treatment had a significant impact on the thawing process, which significantly shortened the time required for beef to pass through the largest ice crystal zone. This phenomenon shows that DBD treatment can effectively accelerate the thawing process of beef and make it reach the appropriate temperature range faster. Under the condition of applying voltage, DBD can transfer some energy to the surface of beef, which is absorbed by water molecules in beef, destroying its original hydrogen bond structure, promoting the transformation of water molecules from large crystal form to small crystal form, and then accelerating the liquefaction process of water. At the same time, the ion wind produced in the DBD process plays a key role in the thawing effect. The ion wind forms turbulence and vortex on the surface of beef, which significantly improves the heat transfer efficiency and makes it easier for water molecules to absorb heat from the surrounding environment, thus accelerating the thawing speed of beef. This phenomenon is supported by Cai et al. [35]. In addition, the thawing time is also affected by the needle distance, which is caused by the principle of electric field superposition. When the needle tip approaches, the change in potential distribution will affect the intensity of the ion wind and then affect the thawing time. On the other hand, it can be seen from Figure 2c,d that with the increase in voltage, the speed of ion wind will also increase accordingly, thus further shortening the thawing time. Compared with corona discharge, DBD technology can generate more plasma and improve the ion wind speed, thus achieving a faster thawing effect. Jung et al. [36] also found that dielectric barrier discharge treatment can promote the temperature of the sample to rise faster, which is consistent with our research conclusion.

### 3.4. WHC

Table 1 shows the WHC of the beef after thawing. Compared with natural thawing, the thawing loss, cooking loss, and centrifugal loss of beef thawed with DBD were significantly reduced (*p* < 0.05). With the increase in voltage and the change in needle distance, all parameters decrease to varying degrees. Too long thawing time in the natural thawing will increase the water loss between muscle fibers, thus increasing the thawing loss. These results showed that DBD thawing had a positive effect on the WHC and could improve the WHC of beef. WHC is closely related to the electrostatic charge effect of protein molecules. When the net charge of protein is zero, the positive and negative charges are equal, and the attraction between positive and negative groups will lead to a decrease in the WHC of protein [37]. At the same time, the repulsive force of myofibril structure is weakened, which leads to the mutual aggregation of myofibril structures, thus making the space inside myofibril more narrow [38]. Xia et al. [24] conducted thawing experiments on pork with water thawing, lotic water thawing, and microwave thawing, and the thawing losses were 4.77%, 5.50%, and 6.64%, respectively. The thawing loss of DBD thawing was between 4.18–5.34% and less than microwave thawing and lotic water thawing. Jia et al. [39] found that although HVEF thawing affected the color of rabbit meat, this method maintained a higher WHC and better texture with reduced denaturation of myofibrillar proteins and some sarcoplasmic proteins compared to still air thawing, resulting in improved WHC. Xu et al. [40] similarly found that HVEF treatment increased the WHC of pork. Therefore, DBD can ionize a lot of charges, thus enhancing the electrostatic repulsion between myofibrils, reducing the damage of beef cells during thawing, preventing water loss, and improving the water retention capacity of thawed beef.

### 3.5. Color

The color of meat is an important factor in meat quality because consumers regard the color of meat as an index to evaluate freshness and quality [41]. Table 2 shows the color changes in beef after thawing with different voltages and needle distances. Compared with the fresh meat samples, the *L* value decreased significantly after thawing in all experimental groups. There was a high correlation between the *L* value and moisture content, and the evaporation of a small amount of moisture from the beef surface can account for the decrease in the *L* value after thawing [42]. Due to the action of the ionic wind, the ΔL* value of beef treated by DBD decreased significantly. Kim et al. [43] also came to the same conclusion when studying the DBD system for processing pig loins. The redness value of meat is mainly determined by the content, molecular type, and chemical state of myoglobin [41]. With increasing voltage, the Δa* value first increases and then decreases. In the natural thawing, with the extension of thawing time, the meat surface moisture loss and drying. Some of the oxymyoglobin forms the metmyoglobin, which causes the Δa* value to decrease. In DBD treatment, the myoglobin from the meat surface combines with oxygen in the air to produce oxymyoglobin, thereby significantly increasing Δa*. However, with the continuous increase in voltage, strong oxidizing substances (such as reactive oxygen species, reactive nitrogen, ozone, etc.) produced by DBD will accelerate the oxidation of myoglobin and oxymyoglobin and form methemoglobin, leading to the browning of the beef surface and decreasing the a* value. This is why the value of beef increased significantly after DBD thawing, but there was no obvious change after natural thawing. Compared with the natural thawing, the Δb* value of beef thawed by DBD increased significantly. Meanwhile, the Δ*E* is affected by ΔL* and Δa* values, and the experimental groups have obvious differences. Compared with the natural thawing, the Δ*E* of the beef thawed by DBD is higher, indicating that the DBD thawing can change the brightness and redness of the beef. Meanwhile, the beef in the DBD treatment had lower L*, higher a*, and similar b*. Increased voltage results in significantly increased ΔL*, indicating that the voltage remarkably influences the L* of the beef surface. It indicated that the voltage had a great influence on the lightness of the beef surface. When there is an increase in voltage, the dielectric barrier discharge produces an enhanced ionic wind, which causes the surface of the beef to become dehydrated. The strong oxidation of plasma reduces the ability to reduce the metmyoglobin to the myoglobin, leading to browning on the surface of beef and reducing the brightness [38]. Therefore, DBD thawing technology has a certain effect on the color change in beef.

### 3.6. Protein Analysis

**Figure 5:**
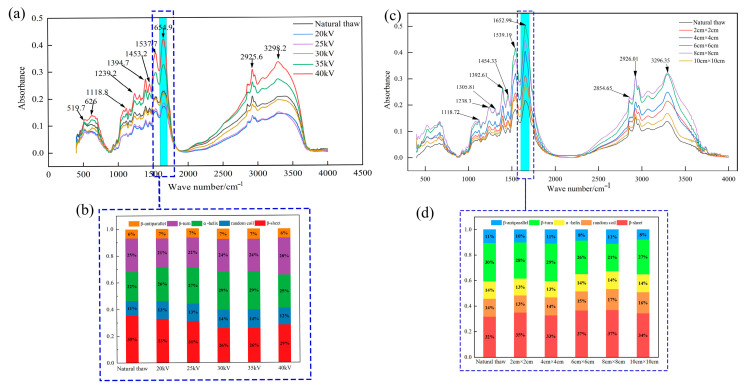
Fourier infrared spectra and the proportion of protein secondary structure of beef thawed by DBD. (**a**,**b**) are infrared spectrum and protein secondary structure at different voltages, respectively; (**c**,**d**) are infrared spectrum and protein secondary structure at different needle distances, respectively.

Figure 5a,c show Fourier infrared spectra of thawed beef. There are many characteristic peaks in the overlapping FTIR spectra of thawed beef. COO symmetrically extends the absorption peak around 1393 cm^−1^, C-O stretching and C-H bending vibration around 1118 cm^−1^, and the bending mode of C-O-H and methyl group around 1454 cm^−1^. Amides I, II, and III appear at the absorption peaks around 1653 cm^−1^, 1538 cm^−1^, 1238 cm^−1^, and 1305 cm^−1^, respectively. The symmetric stretching and asymmetric stretching of CH_2_ appear at the absorption peak around 2854 cm^−1^ and 2926 cm^−1^, respectively, and the N-H stretching of protein appears at the absorption peak around 3296 cm^−1^ [44]. When comparing the absorption intensity of characteristic peaks, it was found that the positions of characteristic peaks of beef thawed by DBD remained consistent. The results showed that the protein structure of beef after DBD thawing did not change substantially, and no new chemical bonds were formed. However, it is worth noting that the intensities of these absorption peaks are different. Specifically, the absorption peak intensity of beef treated by DBD technology is significantly enhanced, which implies the excellent ability of DBD treatment to retain the internal compounds and functional groups of beef and thus helps to maintain the nutritional components of beef to the greatest extent. The thawing effect of different voltages and needle distances is obviously different. When the voltage is 40 kV and the needle spacing is 8 cm × 8 cm, the absorption peak intensity is the highest and the thawing effect is the best.

In order to explore the secondary structure changes in beef protein after thawing, the 1600–1700 cm^−1^ band of the Fourier infrared spectrum was selected for detailed analysis. By deconvolution, second derivative, and curve fitting, the relative content of the secondary structure of beef protein in this band under different thawing methods was calculated. Different bands correspond to different types of protein secondary structure: β-sheet structure at 1600–1642 cm^−1^ band, as a commonly ordered structure in protein, can maintain the stability of protein; the band of 1642–1650 cm^−1^ is a random curly structure with disordered structure; the band of 1650–1660 cm^−1^ has a highly stable α-helix structure; the band of 1660–1680 cm^−1^ is a β-turns structure which plays a role in connecting different regions; β-antiparallel structure is found in the band of 1680–1700 cm^−1^ [27]. In Figure 4b,d, the relative contents of these protein secondary structures in different thawing groups can be seen. It is worth noting that the content of the secondary structure of each protein in beef has changed significantly after DBD thawing. That is, the content of the α-helix structure increases with the change in voltage, the content of the β-sheet structure increases with the change in needle distance, and the content of the β-turn structure decreases. These changes showed that the content of ordered structures (such as α-helix and β-sheet) increased significantly after thawing, while the content of disordered structures (such as random curl) decreased significantly. This structural change makes the protein more stable, which helps improve the quality indexes of beef such as WHC. In addition, our results are consistent with those of Vahed et al. [45], who used EHD to treat germinated mung beans. They found that after EHD treatment, the random curl and β-sheet structure in mung bean protein also increased. This further proves that the electric field treatment technology has a significant regulatory effect on the secondary structure of proteins. To sum up, our research shows that dielectric barrier discharge thawing technology is an effective method to improve the quality of thawed beef. By regulating the secondary structure of the protein, we can significantly improve the key quality indexes, such as the water-holding capacity of beef.

### 3.7. Statistical Analysis

**Figure 6:**
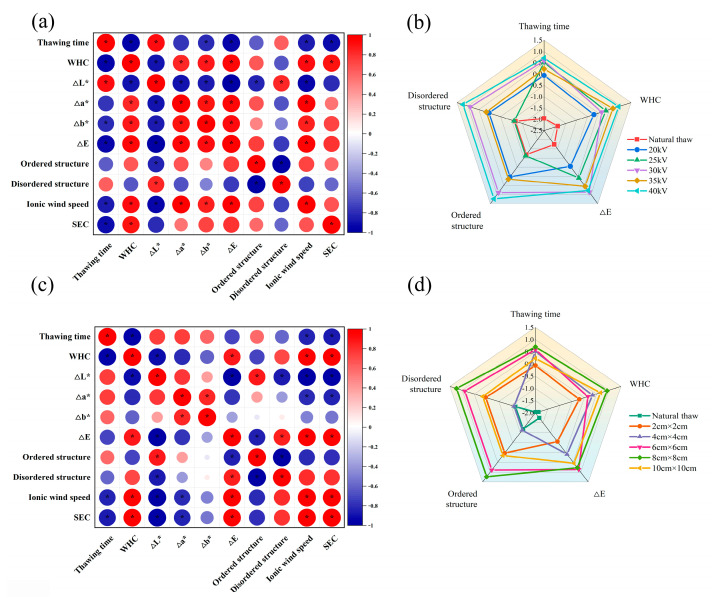
Statistical analyses. (**a**) Pearson’s correlation matrix for thawing at different voltages; (**b**) radar map for thawing at different voltages; (**c**) Pearson’s correlation matrix for thawing at different needle distances; (**d**) radar map for thawing at different needle distances. *: It means that there is a significant difference between the sample mean values (*p* < 0.05).

The obtained experimental data were normalized, and correlation analysis was carried out. Figure 6a,c show the Pearson correlation coefficient matrix between the indexes of the thawing experiment under the influence of different voltages and needle distances. As can be seen from the figure, the thawing time is negatively correlated with ion wind speed and specific energy consumption, and the greater the ion wind speed, the shorter the thawing time. WHC is negatively correlated with thawing time. At the same time, the relationship between WHC and the protein structure of thawed beef was influenced by experimental parameters. When the voltage of DBD thawing is changed, WHC is negatively correlated with the ordered structure of protein and positively correlated with the disordered structure. When the needle distance changes, the correlation is completely opposite. For color components, ΔL* is positively correlated with thawing time and negatively correlated with WHC. Δ*E* is negatively correlated with thawing time and positively correlated with WHC. However, Δa* and Δb* are also affected by experimental parameters and have different correlations. Therefore, the change in experimental parameters significantly affects the correlation of all experimental indexes (*p* < 0.05), which provides a theoretical and experimental basis for exploring the mechanism of beef water retention by dielectric barrier discharge thawing technology. Figure 6b, d show radar images drawn by thawing parameters of beef under different discharge parameters in the DBD system. As can be seen from the figure, the radar area surrounded by various parameters of beef after natural thawing is much smaller than that of DBD thawing. The larger the radar map area, the better the quality of beef. The voltage of 40 kV and the needle distance of 8 cm × 8 cm are the most suitable conditions for thawing beef.

## 4. Conclusions

In the DBD process, increasing the voltage increases the ionic wind speed and changes the plasma composition. Compared with natural thawing, DBD thawing can shorten thawing time and increase WHC, which has the possibility of application in the beef thawing industry. FTIR scanning of beef shows that DBD defrosting can effectively retain nutrients and transform the disordered structure of beef protein secondary structure into an ordered structure. In the experimental group, when the voltage is 40 kV and the needle distance is 8 cm × 8 cm, the thawing time of beef is the shortest, the water retention capacity of thawed beef is better, the color saturation increases the most, and the ingredient retention effect is good. Therefore, the whole quality of thawed beef is the best when the voltage is 40 kV and the needle distance is 8 cm × 8 cm. A technology that moves from the laboratory to industrialization goes through three stages: laboratory research, pilot testing, and industrialization. These experimental results are from the laboratory research stage of DBD thawing technology. The main purpose is to explore the laws of DBD thawing and provide experimental data and a theoretical basis for further research. In the next research step, we will increase the sample weight and conduct pilot tests.

## Figures and Tables

**Table 1:** Water-holding capacity of beef after thawing.

	Thawing Loss (%)	Cooking Loss (%)	Centrifuge Loss (%)
Natural thaw	6.00 ± 0.02 ^d^	49.96 ± 0.69 ^a^	13.05 ± 0.03 ^a^
20 kV	4.79 ± 0.24 ^c^	44.86 ± 0.54 ^b^	9.55 ± 0.85 ^b^
25 kV	4.87 ± 0.03 ^c^	44.60 ± 0.35 ^bc^	10.49 ± 0.41 ^bc^
30 kV	5.42 ± 0.11 ^b^	43.09 ± 0.47 ^bc^	9.49 ± 0.31 ^bc^
35 kV	5.75 ± 0.03 ^ab^	43.02 ± 0.62 ^c^	10.40 ± 0.44 ^c^
40 kV	4.91 ± 0.09 ^a^	43.22 ± 0.22 ^c^	10.17 ± 0.63 ^c^
2 cm × 2 cm	5.34 ± 0.03 ^ab^	44.00 ± 0.07 ^ab^	10.85 ± 0.05 ^ab^
4 cm × 4 cm	4.98 ± 0.05 ^b^	43.18 ± 0.02 ^b^	10.46 ± 0.08 ^ab^
6 cm × 6 cm	4.80 ± 0.05 ^b^	44.79 ± 0.06 ^ab^	9.57 ± 0.08 ^bc^
8 cm × 8 cm	4.18 ± 0.03 ^b^	44.20 ± 0.09 ^ab^	8.58 ± 0.01 ^d^
10 cm × 10 cm	5.10 ± 0.04 ^b^	44.47 ± 0.02 ^ab^	8.14 ± 0.09 ^cd^

Note: Different letters indicate significant differences (*p* < 0.05) between sample means.

**Table 2:** Colors of fresh meat and thawed meat.

	$\Delta L^{*}$	$\Delta a^{*}$	$\Delta b^{*}$	ΔE
Natural thaw	−3.85 ± 0.21 ^a^	1.32 ± 0.53 ^c^	1.35 ± 0.54 ^a^	4.30 ± 0.53 ^e^
20 kV	−3.92 ± 0.43 ^b^	2.54 ± 0.24 ^b^	1.83 ± 0.42 ^a^	5.09 ± 0.12 ^e^
25 kV	−4.27 ± 0.25 ^bc^	3.07 ± 0.15 ^ab^	1.95 ± 0.17 ^a^	5.61 ± 0.34 ^c^
30 kV	−5.19 ± 0.53 ^cd^	3.01 ± 0.45 ^a^	2.17 ± 0.04 ^a^	6.38 ± 0.27 ^a^
35 kV	−6.83 ± 0.21 ^d^	2.90 ± 0.25 ^b^	2.21 ± 0.11 ^a^	7.76 ± 0.20 ^c^
40 kV	−8.12 ± 0.28 ^e^	2.88 ± 0.46 ^c^	2.19 ± 0.06 ^a^	8.88 ± 0.08 ^b^
2 cm × 2 cm	−6.75 ± 0.58 ^b^	1.38 ± 0.21 ^c^	1.72 ± 0.23 ^b^	7.11 ± 0.47 ^d^
4 cm × 4 cm	−7.18 ± 0.71	2.75 ± 0.06 ^b^	2.29 ± 0.38 ^a^	8.61 ± 0.60 ^c^
6 cm × 6 cm	−9.56 ± 0.13 ^e^	3.44 ± 0.23 ^a^	2.37 ± 0.09 ^a^	10.43 ± 0.12 ^a^
8 cm × 8 cm	−9.19 ± 0.37 ^de^	3.81 ± 0.34 ^a^	2.30 ± 0.19 ^a^	10.22 ± 0.19 ^ab^
10 cm × 10 cm	−8.61 ± 0.38 ^cd^	3.48 ± 0.24 ^a^	2.72 ± 0.27 ^a^	9.69 ± 0.28 ^b^

Note: Different letters indicate significant differences (*p* < 0.05) between sample means.

## Data Availability

The original contributions presented in the study are included in the article, further inquiries can be directed to the corresponding authors.
